# Application of a transparent artificial intelligence algorithm for US adults in the obese category of weight

**DOI:** 10.1371/journal.pone.0304509

**Published:** 2024-05-31

**Authors:** Alexander A. Huang, Samuel Y. Huang

**Affiliations:** 1 Northwestern University Feinberg School of Medicine, Chicago, Illinois, United States of America; 2 Virginia Commonwealth University School of Medicine, Richmond, Virginia, United States of America; University of Montenegro-Faculty of Medicine, MONTENEGRO

## Abstract

**Objective and aims:**

Identification of associations between the obese category of weight in the general US population will continue to advance our understanding of the condition and allow clinicians, providers, communities, families, and individuals make more informed decisions. This study aims to improve the prediction of the obese category of weight and investigate its relationships with factors, ultimately contributing to healthier lifestyle choices and timely management of obesity.

**Methods:**

Questionnaires that included demographic, dietary, exercise and health information from the US National Health and Nutrition Examination Survey (NHANES 2017–2020) were utilized with BMI 30 or higher defined as obesity. A machine learning model, XGBoost predicted the obese category of weight and Shapely Additive Explanations (SHAP) visualized the various covariates and their feature importance. Model statistics including Area under the receiver operator curve (AUROC), sensitivity, specificity, positive predictive value, negative predictive value and feature properties such as gain, cover, and frequency were measured. SHAP explanations were created for transparent and interpretable analysis.

**Results:**

There were 6,146 adults (age > 18) that were included in the study with average age 58.39 (SD = 12.94) and 3122 (51%) females. The machine learning model had an Area under the receiver operator curve of 0.8295. The top four covariates include waist circumference (gain = 0.185), GGT (gain = 0.101), platelet count (gain = 0.059), AST (gain = 0.057), weight (gain = 0.049), HDL cholesterol (gain = 0.032), and ferritin (gain = 0.034).

**Conclusion:**

In conclusion, the utilization of machine learning models proves to be highly effective in accurately predicting the obese category of weight. By considering various factors such as demographic information, laboratory results, physical examination findings, and lifestyle factors, these models successfully identify crucial risk factors associated with the obese category of weight.

## Introduction

Obesity is a worldwide concern, including in the United States, where it has reached epidemic proportions. Over the past few decades, general epidemiological trends indicate a steady increase in the prevalence of obesity across all age groups and socioeconomic strata. This surge in obesity rates has far-reaching consequences for public health, as it is associated with a myriad of serious health issues and imposes a significant economic burden on individuals and society [1].

The alarming rise of obesity in the American population has raised numerous red flags for policymakers, healthcare providers, and researchers. Obesity is not just a matter of aesthetics or body image; it is a multifaceted problem with severe health implications. Individuals affected by obesity are at a higher risk of developing chronic conditions such as type 2 diabetes, cardiovascular diseases, hypertension, and certain cancers [1–4]. Moreover, obesity has been linked to reduced quality of life, decreased life expectancy, and increased healthcare costs.

The biochemical pathways that underlie obesity are complex and multifactorial. Genetic predisposition, environmental factors, sedentary lifestyles, and poor dietary choices all play pivotal roles in the development and progression of obesity. Understanding these underlying mechanisms is crucial for designing effective interventions and tailored treatment strategies.

In response to the obesity epidemic, health authorities have established guidelines and recommendations for its prevention and management. These guidelines typically emphasize a comprehensive approach that includes dietary modifications, increased physical activity, behavior change strategies, and, in some cases, medical interventions. Nevertheless, combating obesity remains a challenge due to its multifaceted nature and the need for personalized interventions [2].

Shapely Additive Explanations (SHAP) has emerged as a promising tool for understanding the complex interplay of factors contributing to obesity. SHAP explanations provide transparent and interpretable insights into the machine learning models used to predict obesity and help identify the most influential features driving the predictions. This enhances our understanding of the complex relationships between various factors and the obese category of weight [5, 6].

The aim of this study is to leverage the power of machine learning, specifically XGBoost, along with SHAP explanations to improve the prediction of the obese category of weight. By utilizing data from the US National Health and Nutrition Examination Survey (NHANES), we seek to investigate the associations between obesity and various demographic, dietary, exercise, and health factors. The ultimate goal is to gain deeper insights into obesity’s underlying mechanisms, contribute to the development of more effective preventive strategies, and facilitate timely and personalized management of obesity-related health conditions. By addressing obesity at its root causes, we hope to pave the way for a healthier and more resilient population.

## Methods

A cross-sectional cohort study was conducted with participants who responded to a detailed questionnaire covering demographic information, dietary habits, exercise routines, mental health, as well as laboratory tests and physical examinations using data from the publicly available National Health and Nutrition Examination Survey (NHANES). The National Center for Health Statistics’ (NCHS) Ethics Review Board gave its approval for the study’s data gathering and processing. All data, including medical records, survey responses, and demographic data, were de-identified before analysis to safeguard the participants’ confidentiality and privacy. Participants gave written agreement prior to the study’s start allowing the public release of their data.

### Dataset and cohort selection

The National Center for Health Statistics (NCHS) developed the National Health and Nutrition Examination Survey (NHANES) 2017–2020 to evaluate the health and nutritional status of the American population. The Centers for Disease Control and Prevention (CDC) conducted a comprehensive series of cross-sectional, multi-stage surveys to collect data on health, nutrition, and physical activity for the NHANES dataset. Our investigation focused on adult participants (aged 18 and above) in the NHANES dataset who completed demographic, dietary, exercise, and mental health questionnaires, as well as underwent physical and laboratory examinations. This sample was selected to represent the national population of the United States.

### Assessment of obesity

The obese category of weight is defined as having a Body Mass Index (BMI) greater than or equal to 30. BMI is calculated by dividing an individual’s weight (in kilograms) by the square of their height (in meters). A BMI of 30 or above indicates obesity, as per the standard classification set by the World Health Organization (WHO) and many health authorities.

### Model construction and statistical analysis

In this study, the NHANES dataset encompassed covariates that involved socioeconomics, dietary data, actual assessments, research center outcomes, and clinical surveys. Univariate analysis was initially used to explore the relationship between these covariates and the obese category of weight, the outcome variable. To identify strong independent covariates, the machine learning model selected variables with p-values below 0.0001 from the univariate analysis before considering their interaction in the larger model. XGBoost, a widely used and effective algorithm in healthcare predictions, was chosen for this review. Past studies using NHANES data have identified XGBoost as the optimal algorithm, offering a balance of training efficiency, model accuracy, and interpretability. The dataset was split into a train set (80%) and a test set (20%) to determine the parameters for the final model fit. Model performance was evaluated using various parameters, including the area under the receiver operator characteristic curve (AUROC), sensitivity, specificity, positive predictive value, negative predictive value, prevalence, detection rate, detection prevalence, and balanced accuracy. These parameters were used to assess and evaluate the model’s performance. Additionally, before carrying out the XGBoost modeling that was present in this paper, other machine learning methods just as artificial neural networks, gradient boost modeling, and random forest, to name a few, were utilized. XGBoost was the most accurate by all metrics (AUROC, sensitivity, specificity) combined and thus was utilized in this paper.

### Model feature importance statistics and SHAP visualization

The Gain metric assesses a feature’s importance in the model by calculating its individual contribution for each tree. A higher Gain value suggests greater significance in generating predictions compared to other features. On the other hand, the Cover metric indicates the relative number of observations associated with a specific feature. It is calculated by summing up the occurrences of that feature across all trees. For example, if feature one appears in 15, 10, 8, and 5 observations in tree one, tree two, tree three, and tree four, respectively, the Cover metric for feature one would be 38 observations. The Cover metric is then expressed as a percentage based on the total cover for all features. Additionally, the Frequency metric represents the relative occurrence of a particular feature in the model’s trees. Using the previous example, if feature one appears in 3, 2, 4, and 1 splits within tree one, tree two, tree three, and tree four, respectively, the weightage for feature one would be 10. The Frequency for feature one is determined by calculating its percentage weight relative to the weights of all features.

## Results

Table 1 shows the 6,146 patients that met the inclusion criteria in this study. The average age was 58.39 (SD = 12.94). Individuals had mean HS C-Reactive Protein levels of 4.34 mg/L (SD = 8.89), insulin levels of 15.13 umol/mL (SD = 25.09), Blood lead levels of 1.33 ug/dL (SD = 1.30), Blood cadmium levels of 0.50ug/L (SD = 0.56), Uric acid levels of 5.48 mg/dL (SD = 1.48), Creatinine levels of 121.99 mg/dL (SD = 80.62). Compared to those in the obese category of weight to those that were not, there was a mean HS C-Reactive Protein levels of 5.89 mg/L (SD = 9.56) compared to a mean HS C-Reactive Protein levels of 3.14 mg/L (SD = 8.14), insulin levels of 21.85 umol/mL (SD = 33.16) compared to insulin levels of 10.21 umol/mL (SD = 15.11), Blood lead levels of 1.15 ug/dL (SD = 1.29) compared to Blood lead levels of 1.47 ug/dL (SD = 1.30), Blood cadmium levels of 0.43ug/L (SD = 0.54) compared to Blood cadmium levels of 0.55ug/L (SD = 0.56), Uric acid levels of 5.80 mg/dL (SD = 1.51) compared to Uric acid levels of 5.24 mg/dL (SD = 1.42), Creatinine levels of 134.34 mg/dL (SD = 84.15) compared to Creatinine levels of 112.60 mg/dL (SD = 76.52).

**Table 1:** Demographic variables.

Total	All Individuals	Obese	Not Obese	
Total	6146	2625	3521	
Age (years)	58.39 (12.94)	57.42 (12.43)	59.12 (13.27)	p<0.0001
Gender				
Female	3122 (0.51)	1464 (0.55)	1678 (0.48)	p<0.0001
Male	3024 (0.49)	1181 (0.45)	1827 (0.52)	p<0.0001
Race/ethnicity				
Non-Hispanic White	2252 (0.37)	945 (0.36)	1307 (0.37)	0.6281
Non-Hispanic Black	1636 (0.27)	866 (0.33)	770 (0.22)	p<0.0001
Hispanic	1257 (0.2)	578 (0.22)	679 (0.19)	0.0741
Other	1001 (0.16)	236 (0.08)	765 (0.22)	p<0.0001
Creatinine, urine (mg/dL)	121.99 (80.62)	134.34 (84.15)	112.60 (76.52)	p<0.0001
Creatinine, urine (umol/L)	10784.10 (7126.76)	11875.98 (7438.47)	9953.46 (6764.21)	p<0.0001
Urinary Arsenous acid (ug/L)	0.29 (0.41)	0.24 (0.36)	0.32 (0.44)	p<0.0001
Urinary Monomethylarsonic acid (ug/L)	0.50 (0.60)	0.42 (0.51)	0.56 (0.65)	p<0.0001
Direct HDL-Cholesterol (mmol/L)	1.40 (0.42)	1.29 (0.35)	1.48 (0.45)	p<0.0001
Triglyceride (mg/dL)	114.94 (97.10)	126.47 (110.39)	106.50 (85.12)	p<0.0001
Triglyceride (mmol/L)	1.30 (1.10)	1.43 (1.25)	1.20 (0.96)	p<0.0001
Total Cholesterol (mg/dL)	189.33 (41.85)	186.59 (41.32)	191.45 (42.14)	p<0.0001
Total Cholesterol (mmol/L)	4.90 (1.08)	4.83 (1.07)	4.95 (1.09)	p<0.0001
White blood cell count (1000 cells/uL)	7.20 (5.65)	7.64 (8.06)	6.86 (2.51)	p<0.0001
Monocyte percent (%)	8.29 (2.30)	8.15 (2.12)	8.41 (2.43)	p<0.0001
Monocyte number (1000 cells/uL)	0.58 (0.22)	0.60 (0.20)	0.56 (0.23)	p<0.0001
Segmented neutrophils num (1000 cell/uL)	4.16 (1.70)	4.40 (1.78)	3.98 (1.62)	p<0.0001
Eosinophils number (1000 cells/uL)	0.20 (0.16)	0.21 (0.16)	0.19 (0.16)	p<0.0001
Mean cell volume (fL)	88.97 (6.20)	87.94 (6.13)	89.75 (6.14)	p<0.0001
Red cell distribution width (%)	13.99 (1.38)	14.22 (1.43)	13.81 (1.31)	p<0.0001
Platelet count (1000 cells/uL)	241.76 (65.70)	247.55 (67.91)	237.32 (63.60)	p<0.0001
Mean platelet volume (fL)	8.26 (0.91)	8.34 (0.90)	8.20 (0.91)	p<0.0001
Cotinine, Serum (ng/mL)	56.21 (133.00)	48.46 (126.35)	62.19 (137.63)	p<0.0001
Hydroxycotinine, Serum (ng/mL)	23.75 (66.81)	19.24 (62.54)	27.22 (69.74)	p<0.0001
Serum total folate (ng/mL)	18.38 (12.69)	17.44 (13.71)	19.12 (11.77)	p<0.0001
5-Methyl-tetrahydrofolate (nmol/L)	38.69 (23.88)	36.28 (23.47)	40.60 (24.04)	p<0.0001
HS C-Reactive Protein (mg/L)	4.34 (8.89)	5.89 (9.56)	3.14 (8.14)	p<0.0001
Mercury, methyl (ug/L)	1.23 (2.25)	0.95 (1.46)	1.46 (2.69)	p<0.0001
Insulin (μU/mL)	15.13 (25.09)	21.85 (33.16)	10.21 (15.11)	p<0.0001
Insulin (pmol/L)	90.79 (150.55)	131.09 (198.94)	61.26 (90.68)	p<0.0001
Iron frozen, Serum (ug/dL)	85.50 (34.49)	79.71 (32.67)	89.97 (35.20)	p<0.0001
UIBC, Serum (umol/L)	42.17 (11.19)	43.58 (10.88)	41.08 (11.30)	p<0.0001
Transferrin Saturation (%)	27.27 (11.30)	25.24 (10.39)	28.84 (11.72)	p<0.0001
Blood lead (ug/dL)	1.33 (1.30)	1.15 (1.29)	1.47 (1.30)	p<0.0001
Blood lead (umol/L)	0.06 (0.06)	0.06 (0.06)	0.07 (0.06)	p<0.0001
Blood cadmium (ug/L)	0.50 (0.56)	0.43 (0.54)	0.55 (0.56)	p<0.0001
Blood cadmium (nmol/L)	4.43 (4.94)	3.84 (4.81)	4.89 (4.99)	p<0.0001
Blood mercury, total (ug/L)	1.46 (2.60)	1.14 (1.65)	1.71 (3.12)	p<0.0001
Blood mercury, total (nmol/L)	7.29 (13.00)	5.69 (8.25)	8.53 (15.59)	p<0.0001
Fasting Glucose (mg/dL)	117.65 (40.76)	124.56 (44.12)	112.60 (37.35)	p<0.0001
Fasting Glucose (mmol/L)	6.53 (2.26)	6.91 (2.45)	6.25 (2.07)	p<0.0001
Albumin, refrigerated serum (g/dL)	4.03 (0.33)	3.94 (0.32)	4.09 (0.32)	p<0.0001
Albumin, refrigerated serum (g/L)	40.26 (3.30)	39.45 (3.20)	40.88 (3.23)	p<0.0001
Bicarbonate (mmol/L)	25.59 (2.45)	25.34 (2.49)	25.77 (2.40)	p<0.0001
Globulin (g/dL)	3.10 (0.45)	3.15 (0.44)	3.06 (0.46)	p<0.0001
Glucose, refrigerated serum (mmol/L)	5.89 (2.24)	6.17 (2.42)	5.67 (2.07)	p<0.0001
Iron, refrigerated serum (ug/dL)	86.15 (34.36)	80.34 (32.56)	90.64 (35.05)	p<0.0001
Lactate Dehydrogenase (LDH) (IU/L)	161.56 (35.64)	163.32 (37.29)	160.20 (34.24)	p<0.0001
Osmolality (mmol/Kg)	282.10 (5.75)	282.55 (5.75)	281.75 (5.72)	p<0.0001
Total Bilirubin (umol/L)	7.90 (4.58)	7.42 (4.24)	8.27 (4.80)	p<0.0001
Total Calcium (mg/dL)	9.27 (0.39)	9.24 (0.40)	9.29 (0.38)	p<0.0001
Total Calcium (mmol/L)	2.32 (0.10)	2.31 (0.10)	2.32 (0.10)	p<0.0001
Cholesterol, refrigerated serum (mg/dL)	189.64 (41.88)	186.87 (41.36)	191.79 (42.16)	p<0.0001
Cholesterol, refrigerated serum (mmol/L)	4.90 (1.08)	4.83 (1.07)	4.96 (1.09)	p<0.0001
Total Protein (g/dL)	7.13 (0.46)	7.10 (0.44)	7.15 (0.47)	p<0.0001
Triglycerides, refrig serum (mg/dL)	144.36 (107.87)	155.77 (110.40)	135.52 (105.05)	p<0.0001
Triglycerides, refrig serum (mmol/L)	1.63 (1.22)	1.76 (1.25)	1.53 (1.19)	p<0.0001
Uric acid (mg/dL)	5.48 (1.48)	5.80 (1.51)	5.24 (1.42)	p<0.0001
Uric acid (umol/L)	326.02 (88.26)	344.75 (89.53)	311.51 (84.47)	p<0.0001
N-ace-S-(3,4-dihidxybutl)-L-cys(ng/mL)	424.56 (302.67)	460.12 (317.06)	398.31 (288.94)	p<0.0001
LBX2DF - Blood 2,5-Dimethylfuran (ng/mL)	0.03 (0.07)	0.02 (0.05)	0.03 (0.08)	p<0.0001
LBXVBZ - Blood Benzene (ng/mL)	0.07 (0.17)	0.05 (0.11)	0.08 (0.21)	p<0.0001
LBXVFN - Blood Furan (ng/mL)	0.03 (0.03)	0.03 (0.03)	0.03 (0.04)	p<0.0001
LBXVIBN - Blood Isobutyronitrile (ng/mL)	0.04 (0.05)	0.03 (0.02)	0.04 (0.06)	p<0.0001
Income_Poverty_Ratio	2.70 (1.63)	2.62 (1.62)	2.76 (1.64)	p<0.0001

Descriptive statistics for demographic characteristics and all covariates within the machine learning model, stratified by being in the obese category of weight.

**Fig 1:**
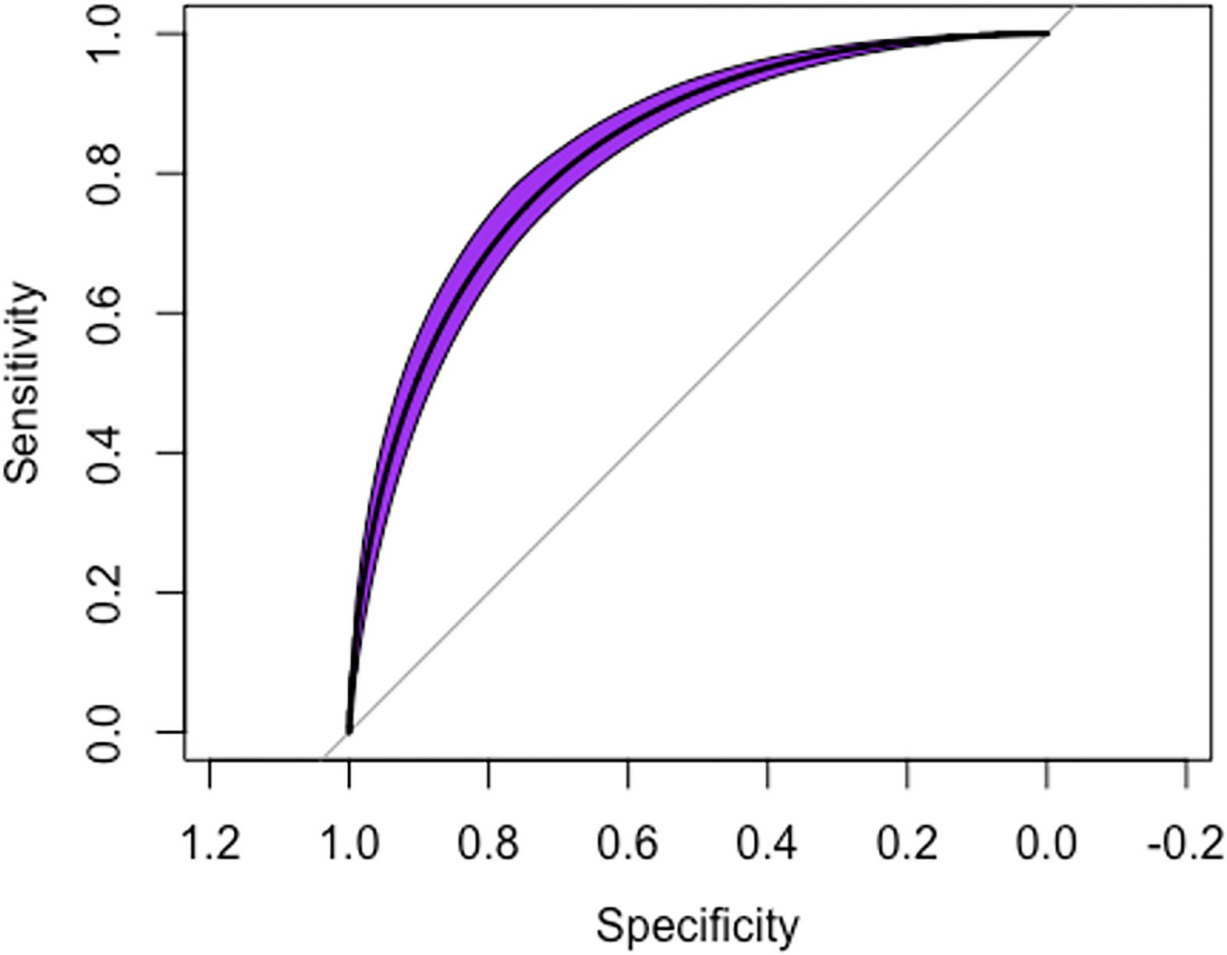
Receiver operator characteristic curve and model statistics. The Receiver operating characteristic curve for the machine-learning model predicting whether the patient were in the obese category of weight. AUROC = 0.8295 (P<0.0001).

The machine learning model had 78 features that were found to be significant on univariate analysis (P<0.0001 used). These were fitted into the XGBoost model, Fig 1 and Table 2 shows an AUROC = 0.8295, Sensitivity = 0.9125, Specificity = 0.5615, Positive predictive value 0.4226, negative predictive value of 0.9481 were observed.

**Table 2:** 

Metric	Value
Sensitivity	0.9125
Specificity	0.5615
Positive Predictive Value (Precision)	0.4226
Negative Predictive Value	0.9481
Prevalence	0.2602
Detection Rate (True Positive Rate)	0.2374
Detection Prevalence	0.5618
Balanced Accuracy	0.737

Table 2 displays the statistical model metrics evaluated by sensitivity, specificity, positive predictive value (PPV), negative predictive value (NPV), Prevalence, Detection Rate, Detection Prevalence, and Balanced Accuracy.

Table 3 shows that the top four covariates ranked by the gain, a measure of the percentage contribution of the covariate to the overall model prediction, were HS C-Reactive Protein (mg/L) (Gain = 0.181), Insulin (uU/mL) (Gain = 0.149), Blood lead (ug/dL) (Gain = 0.056), blood cadmium (ug/L) (Gain = 0.038).

**Table 3:** Model gain statistics.

Feature	Gain	Cover	Frequency
HS C-Reactive Protein (mg/L)	0.181	0.103	0.049
Insulin (μU/mL)	0.149	0.118	0.059
Blood lead (ug/dL)	0.056	0.047	0.046
Blood cadmium (ug/L)	0.038	0.039	0.037
Uric acid (mg/dL)	0.037	0.054	0.042
Creatinine, urine (mg/dL)	0.036	0.041	0.049
MCQ366c - Doctor told you to reduce salt in diet	0.035	0.041	0.015
Direct HDL-Cholesterol (mmol/L)	0.032	0.035	0.032
Albumin, refrigerated serum (g/dL)	0.030	0.042	0.028
Red cell distribution width (%)	0.029	0.039	0.032
Age	0.028	0.039	0.033
Total Protein (g/dL)	0.024	0.035	0.031
Mean platelet volume (fL)	0.017	0.022	0.025
Mean cell volume (fL)	0.017	0.014	0.026
Glucose, refrigerated serum (mmol/L)	0.016	0.021	0.023
White blood cell count (1000 cells/uL)	0.014	0.015	0.023
Lactate Dehydrogenase (LDH) (IU/L)	0.014	0.015	0.027
Income_Poverty_Ratio	0.014	0.010	0.028
Urinary Monomethylarsonic acid (ug/L)	0.014	0.026	0.018
MCQ160a - Doctor ever said you had arthritis	0.014	0.026	0.011
UIBC, Serum (umol/L)	0.009	0.007	0.017
Platelet count (1000 cells/uL)	0.009	0.009	0.019
Serum total folate (ng/mL)	0.009	0.006	0.018
Blood mercury, total (ug/L)	0.008	0.012	0.014
Mercury, methyl (ug/L)	0.008	0.008	0.013
Gender	0.008	0.017	0.009
Monocyte percent (%)	0.008	0.006	0.017
Cotinine, Serum (ng/mL)	0.007	0.005	0.012
Triglycerides, refrig serum (mg/dL)	0.007	0.005	0.014
Total Cholesterol (mg/dL)	0.007	0.006	0.015
MCQ560 - Ever had gallbladder surgery?	0.007	0.010	0.006
Cholesterol, refrigerated serum (mg/dL)	0.006	0.006	0.011
Fasting Glucose (mg/dL)	0.006	0.004	0.012
Segmented neutrophils num (1000 cell/uL)	0.006	0.004	0.013
N-ace-S-(3,4-dihidxybutl)-L-cys(ng/mL)	0.006	0.006	0.011
Iron, refrigerated serum (ug/dL)	0.005	0.006	0.010
Bicarbonate (mmol/L)	0.005	0.006	0.012
Osmolality (mmol/Kg)	0.005	0.004	0.011
Triglyceride (mg/dL)	0.005	0.004	0.010
Transferrin Saturation (%)	0.005	0.005	0.009
Iron frozen, Serum (ug/dL)	0.005	0.004	0.010
Hydroxycotinine, Serum (ng/mL)	0.005	0.006	0.007
5-Methyl-tetrahydrofolate (nmol/L)	0.005	0.005	0.008
MCQ371b - Are you now increasing exercise	0.004	0.011	0.006
Monocyte number (1000 cells/uL)	0.004	0.006	0.008
Total Calcium (mg/dL)	0.004	0.003	0.010
MCQ550 - Has DR ever said you have gallstones	0.004	0.006	0.005
Globulin (g/dL)	0.004	0.002	0.008
MCQ300c - Close relative had diabetes?	0.004	0.003	0.005
Eosinophils number (1000 cells/uL)	0.004	0.004	0.007
Total Bilirubin (umol/L)	0.004	0.002	0.007
MCQ300a - Close relative had heart attack?	0.003	0.005	0.005
MCQ300b - Close relative had asthma?	0.003	0.005	0.005
MCQ371c - Are you now reducing salt in diet	0.003	0.002	0.003
LBXVBZ - Blood Benzene (ng/mL)	0.003	0.004	0.005
MCQ010 - Ever been told you have asthma	0.002	0.004	0.003
Urinary Arsenous acid (ug/L)	0.002	0.002	0.004
Basophils number (1000 cells/uL)	0.001	0.001	0.002
LBX2DF - Blood 2,5-Dimethylfuran (ng/mL)	0.001	0.001	0.002
SMDANY - Used any tobacco product last 5 days?	0.001	0.005	0.001
LBXVFN - Blood Furan (ng/mL)	0.001	0.001	0.001
MCQ160b - Ever told had congestive heart failure	0.001	0.001	0.002
SMQ681 - Smoked tobacco last 5 days?	0.001	0.001	0.001
LBXVIBN - Blood Isobutyronitrile (ng/mL)	0.000	0.001	0.001
SMQ690A - Used last 5 days - Cigarettes	0.000	0.000	0.000

The Gain, Cover, and Frequency of all covariates within the XGBoost model. The Gain represents the relative contribution of the feature to the model and is the most important metric of model importance within this study. Covariates ordered according to the Gain statistic.

**Fig 2:**
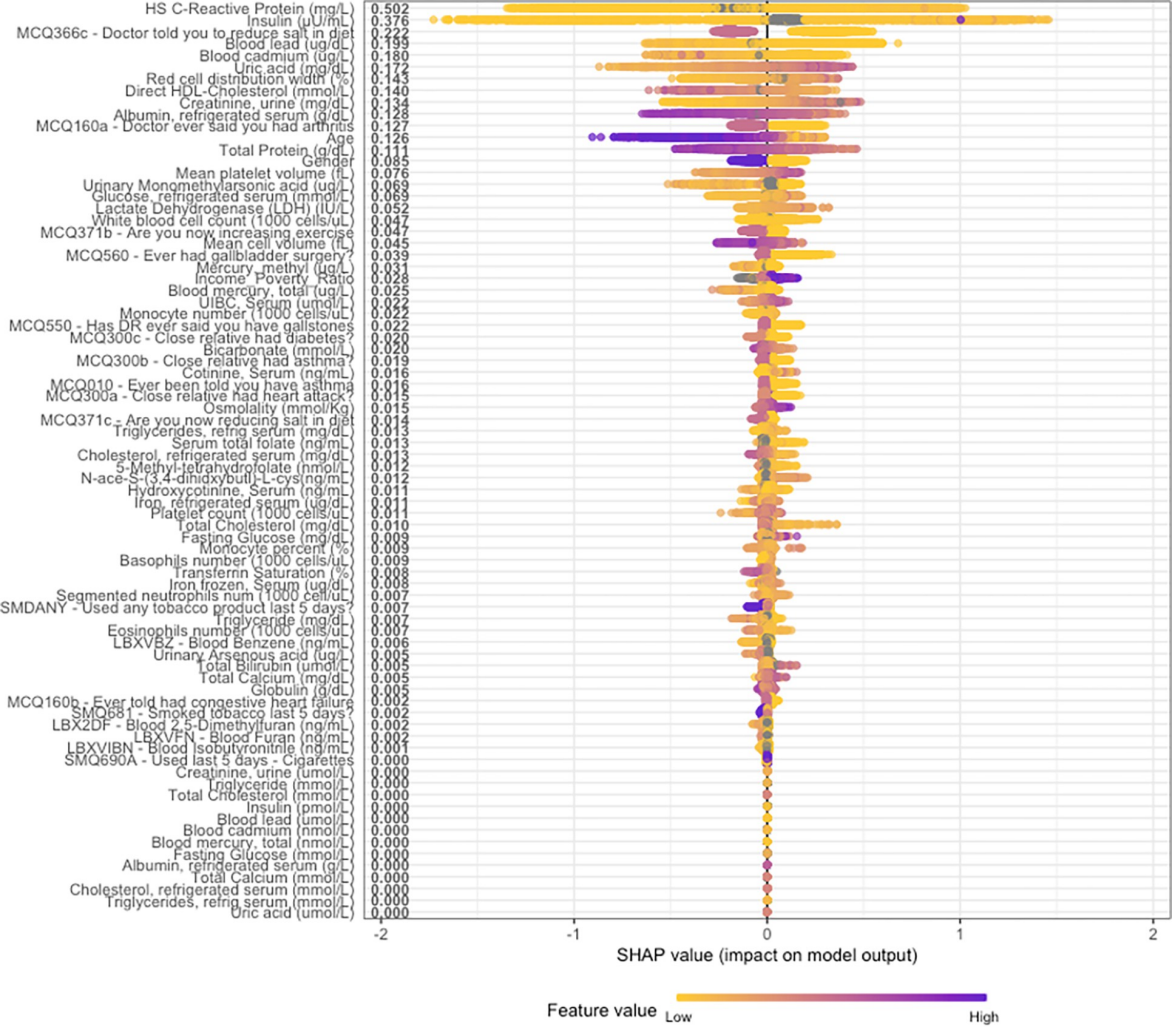
Overall SHAP explanations. SHAP explanations, purple color representing higher values of the covariate while yellow representing lower values of the covariate. X-axis is the change in log-odds for advanced the obese category of weight.

In Fig 2, overall SHAP explanations can be seen for all the statistically significant covariates on univariable regression.

**Fig 3:**
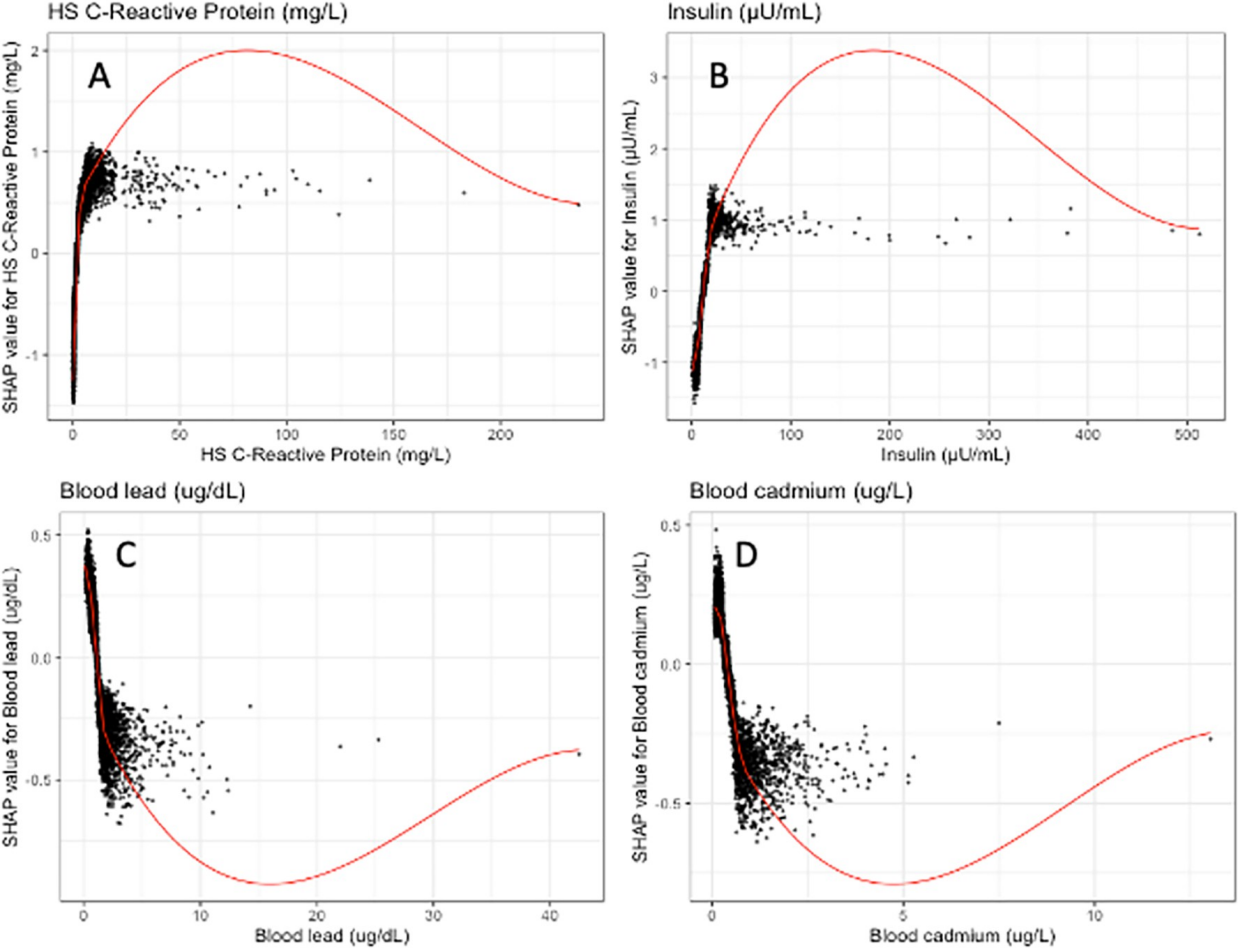
SHAP explanations, covariate value on the x-axis, change in log-odds on the y-axis, red line represents the relationship between the covariate and log-odds for being in the obese category of weight, each black dot represents an observation. Covariates: top left–HS C-Reactive Protein (mg/mL), top right–Insulin (uU/mL), bottom left–Blood lead (ug/dL), bottom right–Blood cadmium (ug/L).

In Fig 3, SHAP visualizations were conducted for the top four continuous covariates by overall SHAP explanations. Trends included a positive association of HS C-reactive Protein and insulin and obesity as well as a negative association between blood lead level and blood cadmium level and obesity.

## Discussion

In this cross-sectional cohort study of US adults, an artificial intelligence algorithm trained on information from the National Health and Nutrition Examination Survey (NHANES) demographic, laboratory, physical examination, and lifestyle factors demonstrated a high predictive accuracy with an area under the receiver operating curve (AUROC) of 0.8295. This indicates that the model was able to strongly predict obesity above what is to be expected of standard chance. The top four covariates that had significant associations with the obese category of weight in the artificial intelligence model based off SHAP value included HS C-Reactive protein, insulin, has doctor told you to reduce salt in the diet and blood lead levels. The top four covariates ranked by gain, which corresponds to the contribution of each feature in the overall artificial intelligence algorithm includes HS C-Reactive Protein (mg/L) (Gain = 0.181), Insulin (uU/mL) (Gain = 0.149), Blood lead (ug/dL) (Gain = 0.056), blood cadmium (ug/L) (Gain = 0.038).

The artificial intelligence algorithm employed in our study demonstrates consistent associations and directionality with those reported in existing literature concerning the obese category of weight [1, 7–9]. These findings, supported by multiple studies, offer valuable insights into how the algorithm perceives these associations. The alignment of our study’s results with established literature enhances our confidence in the algorithm’s ability to accurately capture genuine physiological relationships related to obesity. A notable advantage of the algorithmic approach used in our study is its impartiality in identifying significant covariates. By systematically exploring numerous variables based on mathematical relationships, subjective influence from researchers is minimized. This enables the uncovering of nonlinear patterns, and the covariates can be ranked based on performance metrics, assessing the overall accuracy and reliability of the machine learning model in predicting obesity [10]. SHAP visualizations further aid researchers in comparing their own understanding of variable relationships with the machine learning model’s assessment, allowing for the testing of physiological plausibility. These visualizations provide a valuable tool for validating and comprehending the associations identified by the algorithm, enhancing the interpretability and applicability of the model’s predictions [8].

The study inherits the advantages and limitations associated with cross-sectional multistage survey questionnaire studies. These surveys use multistage sampling techniques, ensuring a representative sample and enabling generalizability to the broader population. However, they offer only a snapshot at a single point in time, restricting the ability to establish causality or temporal sequences. Despite their cost-effectiveness and efficiency in gathering data from a large number of participants within a relatively short period, there is a risk of recall and response bias. Therefore, it is vital to take these limitations into account when interpreting findings from cross-sectional multistage survey questionnaire data. Additionally, multiple variables that are significant may not seem to have causal links, such as lead levels and obesity, and further research is needed to identify if these are just correlations in which the cause is a third-variable (for lead levels and obesity–socioeconomic status), or if there may be causal nature. Further studies are needed to confirm these connections.

## Conclusion

The artificial intelligence algorithm predicted obesity over and above random chance and uncovered associations and relationships in a understandable way for clinicians.

## Abbreviations

NCHS: National Center for Health Statistics
NHANES: National Health and Nutrition Examination Surveys
SHAP: Shapely Additive Explanations
