# Supplementary material for: Application of a transparent artificial intelligence algorithm for US adults in the obese category of weight
Source: PLoS One. 2024 May 31;19(5):e0304509. doi: 10.1371/journal.pone.0304509 (PMC11142543; doi:10.1371/journal.pone.0304509)
Supplement: Attachment — Submitted filename: Reviewer Commments Obesity.docx [file pone.0304509.s001.docx]

5. Review Comments to the Author

Please use the space provided to explain your answers to the questions above. You may also include additional comments for the author, including concerns about dual publication, research ethics, or publication ethics. (Please upload your review as an attachment if it exceeds 20,000 characters)

Reviewer #1: This is a study designed to perdict the obese category of weight and investigate its
relationships with factors, ultimately contributing to healthier lifestyle choices and timely
management of obesity.
The study uses questionnaires that included demographic, dietary, exercise and health information
from the US National Health and Nutrition Examination Survey (NHANES 2017 &ndash; 2020) who had
BMI 30 or higher defined as obesity.
This is an interesting study.
However, I have two questions.

First, the author should address the association between blood lead levels and obesity.
Besides, the author should provide a verification result regarding this algorithm.

**The authors have addressed both of these comments in the paper. First – the blood lead levels and obesity can be associated with socioeconomic status, however, further analysis must be made to understand if this is the correlative third-variable problem. It is difficult to exactly explain away each of the covariates as this is an unbiased data-driven approach rather than one leading to direct discovery of causal linkages.**

**Additionally, verification of the algorithm was attempted through the train-test sets and multiple models executed within this paper. Further research is needed to address this.**

Reviewer #2: In this study, the authors demonstrated the accurate prediction of the obesity category using questionnaires from the US National Health and Nutrition Examination Survery. However, further clarification and revision of the descriptions are needed for publication as follows. First, other machine learning methods, other than XGBoost, need to be investigated to validate the results. Second, the authors need to reorganize the results to make them easier for readers to understand. Please see the following comments to the authors.

Major comments
Methods
1. The authors only used XGBoost to develop the prediction model. Please clarify the rationale for using this model in this study. Also. it would be reasonable to use other machine learning models to validate the results.
2. The authors performed the univariate analysis for each variable. Please provide the name of the statistical analysis for clarification.

1) **The authors have utilized XGBoost based upon its frequency in the machine-learning literature. Additionally, the authros tested multiple different models before choosing xgboost as its final one. The XGBoost model was chosen as it was the strongest. These comments have ben added to the manuscript.**
2) **The authors have added the name of the statistical analysis into the paper.**

Results
1. The authors express the variation of each variable using SD. Does this mean that all variables follow a parametric pattern? If not, please express some of the variables in a non-parametric way. **(Yes, all variables followed a parametric pattern)**
2. Table1 contains so many variables that the readers would be exhausted. The authors express the same variable in a different unit. Please consider choosing either of the units so that the table would be more concise.**(The authors recognize the table-1 is large, however, it is meant for completeness, and different aspects of the table may be of interest to different readers)**
3. Please add the confidence interval to the value of diagnostic accuracy.**(Confidence intervals added throughout the paper)**
4. Figure 2 contains so much information that readers would be confused to interpret the significance of the figure. The lower order of the variables in the SHAP analysis should have less contribution to the model development. Please delete the lower order of the variables to make it more concise. Some of the results could be moved to Supplementary Material. (**This comment is very accurate! We recognize that adding too many variables clutters reading. We recognize these concerns for conciseness of the figure – we include the figure 2 as is since we believe that using human judgment for what contributes an important variable may lead to bias – thus we utilize only algorithmic methodology for variable selection.**
